# Relationships of diabetes and hyperglycaemia with intraocular pressure in a Japanese population: the JPHC-NEXT Eye Study

**DOI:** 10.1038/s41598-020-62135-3

**Published:** 2020-03-24

**Authors:** Akiko Hanyuda, Norie Sawada, Kenya Yuki, Miki Uchino, Yoko Ozawa, Mariko Sasaki, Kazumasa Yamagishi, Hiroyasu Iso, Kazuo Tsubota, Shoichiro Tsugane

**Affiliations:** 1 Department of Ophthalmology, Keio University School of Medicine, 35 Shinanomachi, Shinjuku-ku, Tokyo, 160-8582 Japan; 2 Epidemiology and Prevention Group, Centre for Public Health Sciences, National Cancer Centre, 5-1-1 Tsukiji, Chuo-ku, Tokyo, 104-0045 Japan; 3 Tachikawa Hospital, 4-2-22 Nishiki-cho, Tachikawa, Tokyo, 190-8531 Japan; 4 National Institute of Sensory Organs, National Tokyo Medical Centre, 2-5-1 Higashigaoka, Meguro-ku, Tokyo, 152-8902 Japan; 5 Department of Public Health Medicine, Faculty of Medicine, and Health Services Research and Development Centre, University of Tsukuba, 1-1-1 Tennodai, Tsukuba, Ibaraki, 305-8575 Japan; 6 Ibaraki Western Medical Centre, 555 Otsuka, Chikusei, Ibaraki, 308-0813 Japan; 7 Department of Social and Environmental Medicine, Osaka University Graduate School of Medicine, 2-2 Yamadaoka, Suita, Osaka, 565-0871 Japan

**Keywords:** Glaucoma, Diabetes complications, Risk factors

## Abstract

Although a meta-analysis previously suggested a positive relationship between diabetes and intraocular pressure (IOP), the interrelationships among diabetes, IOP, and other ocular biometric parameters remain unclear. The present study investigated the relationships of diabetes, haemoglobin A1c (HbA1c), and serum glucose with IOP and ocular hypertension (IOP > 21 mmHg) in non-glaucomatous Japanese adults living in Chikusei City. Diabetes was defined as a self-reported history of diabetes, the use of antidiabetic medication, or HbA1c levels ≥6.5%. Among 6,786 enrolled participants aged 40 years and above, 734 were classified as diabetic (10.8%). After adjusting for several confounders, the IOP values were significantly higher in participants with diabetes than in those without diabetes (14.4 ± 0.1 vs. 13.9 ± 0.1 mmHg, P < 0.001) and were also significantly increased in those with elevated HbA1c and serum glucose levels (both P < 0.001). Moreover, diabetes was significantly related to ocular hypertension (multivariable-adjusted odds ratio, 1.75; 95% confidence interval, 1.09–2.81; P < 0.05). The positive influence of diabetes with ocular hypertension was consistent even after adjustment for central corneal thickness. In conclusion, diabetes, elevated HbA1c, and increased serum glucose are significant contributing factors for elevated IOP.

## Introduction

Over the past several decades, the prevalence of type 2 diabetes has increased markedly in Asian countries^1^, and patients with diabetes in these countries currently account for more than 60% of the global diabetic population^2^. Partly due to a marked change in dietary habits and lifestyle in Asia, type 2 diabetes has become a major public health burden, leading to substantial financial loss due to increased morbidity, mortality, and health care expenditures^3^. Diabetes is commonly accompanied by microvascular damage, which might contribute to numerous ocular complications, including elevated intraocular pressure (IOP)^4–6^ and subsequent glaucoma^4,7–10^, a leading cause of irreversible blindness worldwide^11^. According to a recent meta-analysis of 47 studies from 16 countries^9^, a history of diabetes was associated with an average increase of 0.18 mmHg (95% confidence interval [CI], 0.09–0.27) in IOP and a 48% increased relative risk for primary open-angle glaucoma (POAG) compared to the risk in nondiabetic patients.

The underlying mechanisms linking diabetes and elevated IOP have not been fully elucidated. Accumulating evidence suggests that a hyperglycaemic status may disrupt cell and repair functions in the cornea^12–15^, thus potentially affecting the central corneal thickness (CCT)^16,17^. Because IOP values rise by 0.11–1.00 mmHg per 10-μm increase in CCT^18–20^, it is plausible that a positive association between diabetes and high IOP may be partly attributable to a greater CCT in patients with diabetes. Nevertheless, many previous studies that examined the association between diabetes and IOP did not consider CCT measurements^7,21–24^, despite it being a plausible IOP regulating factor^18–20^.

Considerable evidence suggests that systemic and ocular biometric parameters are affected by genetic and environmental factors^9,25–27^. However, population-based studies of such factors in Asians are limited^27–29^. Specifically, in terms of IOP, Japanese adults may have unique characteristics compared to other populations: a relatively low IOP^29–31^ and a significant prevalence of normal-tension glaucoma (3.3–3.6%)^32,33^. The mean IOP in non-glaucomatous Japanese individuals (11.8–14.5 mmHg)^29–31^ was lower than that in Caucasians (17.2 mmHg)^34^ and other Asians, including Malays (15.3 mmHg)^35^ and Indians (15.8 mmHg)^35^. Since the suggested positive relationship between diabetes and IOP was largely based on research conducted in Western countries^9^, this relationship should be re-examined in East Asian populations. Furthermore, it is not clear whether diabetes is a CCT-independent cause of elevated IOPs or whether the observed positive association between diabetes and IOP is merely mediated by CCT increases. To the best of our knowledge, no study has investigated the interrelationships among diabetes, IOP, and CCT in a Japanese population.

Here, the influence of type 2 diabetes, serum glucose, and haemoglobin A1c (HbA1c, which is considered to reflect the average serum glucose level in the previous 2–3 months) levels on IOP was evaluated using a large body of data from ophthalmologically normal Japanese adults who participated in an epidemiological survey conducted in Chikusei City, Japan.

## Results

### Characteristics of systemic and ocular factors in participants with and without diabetes

**Figure 1:**
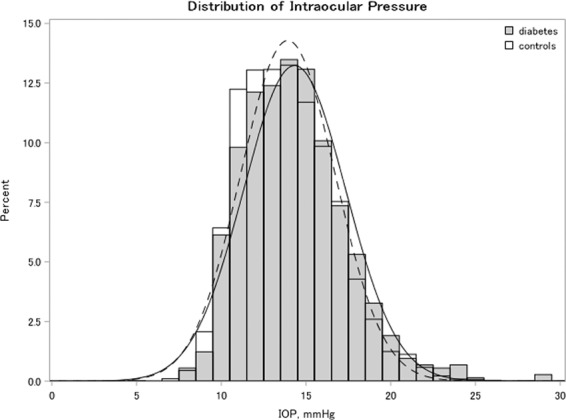
Intraocular pressure (IOP) distribution in the right eye of participants according to their diabetic status. The IOP distribution is indicated by white bars with a dotted line for controls and grey bars with a solid line for patients with diabetes.

Among the 6,786 enrolled participants (4,013 men and 2,773 women), 734 (10.8%; 292 men and 442 women) had diabetes. The mean age ± standard deviation (SD) was slightly higher in patients with diabetes (66.9 ± 7.8 years) than in participants without diabetes (63.3 ± 10.0 years, P < 0.001; Table 1). Individuals with diabetes were more likely to have a history of smoking; higher body mass index (BMI) and waist circumference; higher levels of HbA1c, non-fasting serum glucose, triglycerides, and IOP; and lower high-density lipoprotein cholesterol levels (Table 1). Figure 1 shows the IOP distribution according to the diabetic status. The mean IOP ± SD was 14.3 ± 2.8 mmHg for participants with diabetes and 13.9 ± 2.8 mmHg for those without diabetes (P < 0.001). The demographic breakdown of the IOP data from the participants in this study is presented in Supplementary Table S1. The mean IOP ± SD was 13.7 ± 2.8 mmHg in men and 14.1 ± 2.8 mmHg in women, and the IOP values were inversely correlated with age in both sexes.

**Table 1:** Demographic, systemic, and ocular characteristics of the participants according to their diabetic status^a^.

Characteristics	Diabetes^‡^ (n = 734)	No diabetes (n = 6,052)	P-value
**Demographic features**			
Mean age in years (SD)^b^	66.9 (7.8)	63.3 (10.0)	<0.001
Male participants, n (%)	292 (39.8)	3,721 (61.5)	<0.001
Body mass index, kg/m² (SD)	24.6 (3.8)	23.0 (3.2)	<0.001
Waist circumference, cm (SD)	78.1 (28.6)	74.2 (25.9)	<0.001
Weight, kg (SD)	64.6 (13.1)	57.0 (10.4)	<0.001
Height, cm (SD)	158.6 (10.3)	157.0 (10.1)	<0.001
Smoking status, n (%)			<0.001
Never smokers	36.5	24.2	
Current smokers	16.3	12.6	
Alcohol intake, n (%)			0.01
<23 g/day	56.0	58.3	
23 to <46 g/day	14.0	16.4	
≥46 g/day	30.0	25.2	
**Systemic features**			
Hypertension, n (%)	57.2	44.9	<0.001
Systolic blood pressure, mmHg (SD)	130.3 (17.1)	125.1 (17.6)	<0.001
Diastolic blood pressure, mmHg (SD)	75.3 (11.1)	74.6 (11.6)	0.06
HDL cholesterol, mg/dL (SD)	56.4 (14.2)	64.0 (15.8)	<0.001
LDL cholesterol, mg/dL (SD)	120.3 (32.6)	125.9 (31.1)	<0.001
Triglyceride, mg/dL (SD)^c^	115.0 (77.9)	95.0 (68.5)	<0.001
HbA1c, % (SD)^c^	6.7 (1.2)	5.6 (0.3)	<0.001
Participants with fasting glucose levels, n (%)	452 (61.6)	3,991 (66.0)	0.02
Fasting glucose, mg/dL (SD)^d^	132.4 (33.3)	96.3 (9.5)	<0.001
Non-fasting glucose, mg/dL (SD)^d^	138.2 (45.6)	97.6 (13.1)	<0.001
**Ocular features**			
Central corneal thickness, µm (SD)	554.3 (49.2)	550.0 (59.3)	0.16
Intraocular pressure, mmHg (SD)	14.3 (2.8)	13.9 (2.8)	<0.001
Ocular hypertension, n (%)	24 (3.1)	111 (1.9)	0.01

^a^Values are presented as the means (SDs) for continuous variables and percentages for categorical variables.

^b^Diabetes was defined as self-reported antidiabetic medication use, physician-diagnosed diabetes, or HbA1c ≥ 6.5%.

^b^All values other than age were adjusted for age and sex.

^c^For triglycerides and HbA1c, medians (SDs) are presented.

^d^Serum glucose levels were examined in 2013–2016.

HbA1c, haemoglobin A1c; HDL, high-density lipoprotein; LDL, low-density lipoprotein; SD, standard deviation.

### Relationships of diabetes, haemoglobin A1c, and serum glucose with intraocular pressure

After adjusting for age, sex, and several lifestyle and metabolic factors, diabetes was significantly related to higher IOP values (mean IOP [±standard error, SE] in patients with diabetes vs. participants without diabetes: 14.4 ± 0.1 vs. 13.9 ± 0.1 mmHg, respectively, P < 0.001; Table 2). Similarly, individuals with higher serum HbA1c or serum glucose levels had elevated IOPs (P < 0.001). We confirmed these relationships after controlling for waist circumference, a good proxy for adiposity traits, instead of adjusting for BMI (data not shown), as well as in subgroup analyses by sex (data not shown). Additionally, we further adjusted the relationships for CCT and confirmed that the identified positive relationships of diabetes, serum HbA1c, and serum glucose with IOP were generally consistent.

**Table 2:** Adjusted mean intraocular pressure by diabetes, HbA1c, and serum glucose.

Characteristics	No. (%)	Mean intraocular pressure, mmHg (SE)					
		Age- and sex-adjusted	P-value	Multivariable-adjusted^a^	P-value	Multivariable-adjusted^b^	P-value
**Diabetes**^**c**^							
No	6,052 (89.2)	13.9 (0.1)	<0.001	13.9 (0.1)	<0.001	13.9 (0.1)	<0.001
Yes	734 (10.8)	14.5 (0.1)		14.4 (0.1)		14.3 (0.1)	
**Serum HbA1c, %**							
<6.0%	5,206 (76.7)	13.9 (0.1)	<0.001	13.9 (0.1)	<0.001	13.8 (0.1)	<0.001
≥6.0%	1,580 (23.3)	14.3 (0.1)		14.2 (0.1)		14.1 (0.1)	
**Serum glucose, mg/dL**^**d**^							
Fasting <110 or non-fasting <140	5,282 (86.1)	13.8 (0.1)	<0.001	13.8 (0.1)	<0.001	13.8 (0.1)	<0.001
Fasting ≥ 110 or non-fasting ≥ 140	853 (13.9)	14.6 (0.1)		14.4 (0.1)		14.4 (0.1)	

^a^Adjusted for age, sex, smoking status, alcohol intake, hypertension, and body mass index.

^b^Adjusted for age, sex, smoking status, alcohol intake, hypertension, body mass index, and central corneal thickness.

^c^Diabetes was defined as self-reported antidiabetic medication use, physician-diagnosed diabetes, or HbA1c ≥ 6.5%.

^d^Serum glucose data were only available between 2013–2016 for 6,135 samples out of 6,786 study participants in 2013–2017.

HbA1c, haemoglobin A1c; SE, standard error.

### Relationships of diabetes, haemoglobin A1c, and serum glucose with ocular hypertension

Multivariable-adjusted logistic regression analyses showed that individuals with diabetes had an approximately two-fold higher prevalence of ocular hypertension than those without diabetes (multivariable-adjusted odds ratio [OR], 1.75; 95% CI, 1.09–2.81; P < 0.05; Table 3). Likewise, higher serum HbA1c (≥6.0%) and higher serum glucose (fasting ≥ 110 or non-fasting ≥ 140 mg/dL) levels were significantly related to ocular hypertension (multivariable-adjusted OR [95% CI] for HbA1c ≥ 6.0% vs. <6.0%: 1.47 [1.00–2.17], P < 0.05; for serum glucose with fasting levels ≥110 or non-fasting levels ≥140 mg/dL vs. fasting levels <110 or non-fasting levels <140 mg/dL: 1.80 [1.11–2.92], P < 0.05). Similar relationships were evident after controlling for CCT.

**Table 3:** Multivariable logistic regression analyses of the relationships of ocular hypertension (intraocular pressure > 21 mmHg) with diabetes, HbA1c, and serum glucose.

Characteristics	No. (%)	OR (95% CI)		
		Age- and sex-adjusted	Multivariable-adjusted^a^	Multivariable-adjusted^b^
**Diabetes**^**c**^				
No	112 (1.9)	^1^[Reference]	^1^[Reference]	^1^[Reference]
Yes	23 (3.1)	1.89 (1.19–3.02)^‡^	1.75 (1.09–2.81)^†^	1.92 (1.13–3.28)^†^
**Serum HbA1c, %**				
<6.0%	94 (1.8)	^1^[Reference]	^1^[Reference]	^1^[Reference]
≥6.0%	41 (2.6)	1.59 (1.09–2.32)^†^	1.47 (1.00–2.17)^†^	1.49 (0.95–2.32)
**Serum glucose, mg/dL**^**d**^				
Fasting <110 or non-fasting <140	81 (1.5)	^1^[Reference]	^1^[Reference]	^1^[Reference]
Fasting ≥ 110 or non-fasting ≥ 140	24 (2.8)	1.97 (1.22–3.17)^‡^	1.80 (1.11–2.92)^†^	1.75 (1.07–2.87)^†^

^a^Adjusted for age, sex, smoking status, alcohol intake, hypertension, and body mass index.

^b^Adjusted for age, sex, smoking status, alcohol intake, hypertension, body mass index, and central corneal thickness.

^c^Diabetes was defined as self-reported antidiabetic medication use, physician-diagnosed diabetes, or HbA1c ≥ 6.5%.

^d^Serum glucose data were only available between 2013–2016 for 6,135 samples out of 6,786 study participants in 2013–2017.

Test for significance: ^†^P < 0.05, ^‡^P < 0.01.

CI, confidence interval; HbA1c, haemoglobin A1c; OR, odds ratio.

## Discussion

This large Japanese population-based study assessed the relationships of diabetes, HbA1c, and serum glucose with IOP in non-glaucomatous individuals. After controlling for several possible confounders, the current study suggested that individuals with diabetes had significantly higher IOP than those without diabetes. Furthermore, patients with diabetes had an 80% increased prevalence of ocular hypertension compared to participants without diabetes, even after controlling for CCT measurements.

Consistent with a recent systematic review and meta-analysis^9^, the present results showed that a history of diabetes was related to both increased IOP and the presence of ocular hypertension. Earlier self-reported questionnaire-based studies showed a non-significant association between diabetes and high IOP^36–39^. In the present study, the positive relationship between poor glycaemic control, characterized by increased HbA1c or serum glucose levels, and high IOP was reaffirmed, in addition to the use of a questionnaire-based definition.

Accumulating evidence from epidemiological and animal studies suggests a causal link between hyperglycaemic status and increased IOP^4,6,8,40,41^; however, less is known about how excessive plasma glucose can elevate ocular tension in the anterior chamber. One conceivable explanation may involve the elevation of transforming growth factor (TGF)-β2 levels in the context of diabetes and the subsequent dysfunction of the trabecular meshwork, which reduces the outflow of the aqueous humour and elevates the IOP^41^. TGF-β may inhibit the proliferation and migration of trabecular cells^42–44^. Ochiai *et al*.^45^ and Min *et al*.^46^ reported increased levels of TGF-β2 in the aqueous humour and trabecular meshwork of eyes in individuals with diabetes. At least in mouse studies, human TGF-β2 decreased the aqueous outflow and increased the IOP^41^. Although future studies are warranted, high concentrations of TGF-β2 in diabetes may contribute to increased outflow resistance and elevated IOP. Another publication suggests the accumulation of advanced glycation end-products (AGEs) in trabecular meshwork cells in patients with diabetes^47^. Since increased levels of AGEs induce oxidative stress and apoptosis in human trabecular meshwork cells^48,49^, the impaired trabecular meshwork functions under these conditions may lead to an increased IOP^50^.

The present study provides evidence that the positive relationships between hyperglycaemic status and IOP were significant, even after controlling for CCT. Increases in CCT may lead to overestimated IOP values^35,51,52^, and diabetic patients have relatively greater CCTs due to the osmotic gradients induced by accumulated sorbitol in the cornea^53^. Accordingly, several studies have suggested that a greater CCT may mediate the association between higher glucose levels and elevated IOP^10,54^. In our current study, we tested the potential role of CCT as a mediator of diabetes and IOP. In the multiple linear regression analyses with diabetes predicting CCT (exposure-mediator) and with CCT predicting IOP (mediator-outcome), both relationships were statically significant (P < 0.05). Besides, the diabetes-IOP relationship was also significant after CCT adjustment, suggesting CCT as a partial mediator in our study population^55,56^. These findings are consistent with those of a previous study from Singapore reporting that a higher IOP in patients with diabetes was not primarily mediated by a greater CCT in these patients^10^. Therefore, controlling blood sugar in patients with diabetes is important to reduce future glaucoma susceptibility.

Nevertheless, the link between diabetes and POAG-related traits, including IOP, remains controversial in recent findings from genome-wide association studies^57–59^. Although Shiga *et al*. found a significant positive correlation between diabetes and POAG in a Japanese population^57^, another Japanese genetic study^58^ and a study in Europeans^59^ found no such association. Although genetic analyses are increasingly recognized as useful tools for providing unbiased estimates without confounding by reverse-causality or measurement errors, there are some non-genetic factors (i.e., impaired trabecular meshwork due to hyperglycaemia-induced inflammation or accumulation of AGEs) that possibly explain the link between diabetes and high IOP. Hence, additional large population-based studies examining the complex relationships among diabetes, CCT, and IOP in different ethnic groups are warranted.

This study had several limitations. First, the current findings are based on cross-sectional observations, which prevent the confirmation of causality between an increase or decrease in any of the parameters and changes in IOP. Although future longitudinal studies are warranted, studies assessing the interrelationships between diabetes and ocular biometric parameters, such as the current study, are noteworthy. Second, given that ocular hypertension is diagnosed by comprehensive ocular evaluations, including visual-field tests, the employed IOP measurement by non-contact pneumotonometry is not completely reflective of ocular hypertension. However, the mean IOP ± SD of 14.0 ± 2.8 mmHg in the present study was close to that of 14.5 mmHg in the Tajimi Study^29^, in which 3,021 non-glaucomatous individuals aged >40 years were subjected to Goldmann applanation tonometry. Furthermore, the use of Goldmann applanation tonometry may induce a larger amount of inter-investigator heterogeneity than encountered when using non-contact devices^60^. Generally, hospital-based studies, including detailed ocular investigations, may be hampered by selection bias and power issues. The present large population-based study reduced the effects of these caveats, although the results should still be interpreted cautiously. In addition, we did not include serum glucose levels in our definition of diabetes due to the unavailability of serum glucose levels in 2017. We acknowledge the possible underdiagnosis of diabetes and exposure misclassification. However, we conducted sensitivity analyses for the 2013–2016 participants using a diabetes definition that included serum glucose levels (≥200 mg/dL for non-fasting or ≥126 mg/dL for fasting serum glucose levels) and confirmed that the results were generally consistent (data not shown). Finally, the present data are restricted to a Japanese population, which might affect the generalizability. Nevertheless, the findings can be generally applied to other East Asian populations.

The strengths of our study include adequate power with substantial information on lifestyle and ocular parameters. The ophthalmic databases were rigorously evaluated by experienced ophthalmologists. In the current study, hyperglycaemic status was defined based on both self-reported questionnaires and laboratory data, including serum glucose and HbA1c, which should be more accurate than the data obtained only by self-reported patient information.

In conclusion, we found that diabetes and hyperglycaemia were significantly related to increased IOP among ophthalmologically healthy Japanese adults. This finding underscores the importance of appropriate glycaemic control to avoid IOP-related consequences in patients with diabetes.

## Methods

### Study population

The Japan Public Health Centre-based Prospective Study for the Next Generation (JPHC-NEXT) Eye Study is an ancillary investigation of an ongoing population-based study conducted under the protocol of the JPHC-NEXT Study^61^. Between 2013 and 2017, we performed a systemic and ophthalmological survey, as well as the JPHC-NEXT baseline surveillance in Chikusei City, located in central Japan, approximately 70 km north of Tokyo. All participants were asked about their socio-demographics, lifestyle factors (e.g., alcohol use or smoking status), medical history (e.g., history of diabetes or hypertension), ocular history (i.e., cataract surgery and refractive surgery), and medication use via interviews by trained technicians or via self-administered questionnaires. The health examination included anthropometry, blood pressure, laboratory measurements, and ocular examinations. Of the 9,940 individuals who agreed to participate in the ocular investigations and who were aged over 40 years, we excluded those who had a diagnosis of glaucoma or any history of IOP-lowering treatment (n = 490); a history of ocular laser treatment or surgery, including refractive or cataract surgeries, corneal oedema or dystrophy (n = 1,051); incomplete data on anthropometric or laboratory measurements (n = 1,022); or missing or outlying IOP measurements (n = 591). Thus, 4,013 males and 2,773 females were finally enrolled in this study.

Written informed consent was obtained from all individuals. The institutional review boards at Osaka University, Osaka; National Cancer Center, Tokyo; Keio University, Tokyo; and the University of Tsukuba, Ibaraki approved the study. This study adhered to the tenets of the Declaration of Helsinki and the laws of Chikusei City with respect to maintaining the privacy of the participants.

### Systemic examination and exposure assessment

Anthropometric parameters, including waist circumference, weight, and height, were measured by trained technicians during the study. The BMI was calculated as weight (kg) divided by height (m) squared. Blood pressure was taken on the right upper arm twice by trained technicians with the participant in the sitting position, and the mean value of these two successive readings was utilized in this study. Blood samples were collected for measurements of serum glucose (fasting or non-fasting), HbA1c, and cholesterol concentrations. The fasting state was considered the state after a fasting duration of at least 10 h after the last meal. In 2017, serum glucose was excluded from the examination protocol. Hence, serum glucose data were available only from 2013–2016. Type 2 diabetes was defined as a self-reported history of physician-diagnosed diabetes, self-reported use of antidiabetic medication, or HbA1c levels ≥6.5%^62,63^. In accordance with the current guidelines from the Committee of the Japan Diabetes Society on the Diagnostic Criteria of Diabetes Mellitus^63^, the cut-off points of 6.0% for HbA1c, as well as <110 and <140 mg/dL for fasting and non-fasting serum glucose levels, respectively, were used.

### Ocular examination and assessment of intraocular pressure

Standardised ocular examinations were performed by trained ophthalmologists. The IOP was determined by three successive readings of the right eye obtained by a non-contact tonometer (Tonoref^TM^ II; Nidek Co., Ltd., Tokyo, Japan). Ocular hypertension was defined as a right-eye IOP > 21 mmHg with no optic disc abnormalities, history of self-reported physician-diagnosed glaucoma, or use of any antiglaucoma therapy^64^. CCT was measured in the right eye using a specular-type pachymeter (Specular Microscope XIII; Konan, Nishinomiya, Japan).

### Statistical analysis

Age- and sex-adjusted mean values for continuous variables and proportions for categorical variables were compared according to the participants’ diabetic status using regression analysis and logistic regression analysis, respectively. Analyses of covariance models were used to assess the relationships of mean IOP with diabetes, serum HbA1c, and serum glucose after adjusting for *a priori* known factors that affect IOP, including age (continuous), sex (male vs. female), smoking status (never, ex-smokers, or current smokers), alcohol intake (<23, 23 to <46, ≥46 g/day), hypertension (yes vs. no), and BMI (<25 vs. ≥25 kg/m^2^) in the primary multivariable model. Because CCT may mediate the relationship between hyperglycaemia and IOP, we additionally adjusted for CCT in the secondary model.

Multivariable logistic regression models were used after controlling for the same sets of confounders as stated above to analyse the relationships between the prevalence of ocular hypertension and diabetes, serum HbA1c, and serum glucose. The adjusted ORs and 95% CIs were calculated. All analyses were performed using SAS software (Version 9.4, SAS Institute, Cary, NC, USA). All P-values were two-sided, and a P-value of < 0.05 was considered statistically significant.

## Supplementary information

Supplementary Table S1.

## Data Availability

For information on how to submit an application for gaining access to JPHC-NEXT data and/or biospecimens, please follow the instructions at https://epi.ncc.go.jp/jphcnext/en/access/index.html.
