# Supplementary material for: Relationships of diabetes and hyperglycaemia with intraocular pressure in a Japanese population: the JPHC-NEXT Eye Study
Source: Sci Rep. 2020 Mar 24;10:5355. doi: 10.1038/s41598-020-62135-3 (PMC7093393; doi:10.1038/s41598-020-62135-3)
Supplement: Supplementary file 1 — Supplementary Table S1. [file 41598_2020_62135_MOESM1_ESM.docx]

Supplementary materials

Title: Relationships of diabetes and hyperglycaemia with intraocular pressure in a Japanese population: the JPHC-NEXT Eye Study

Akiko Hanyuda, MD;^1,2^ Norie Sawada, MD, PhD;^2^* Kenya Yuki, MD, PhD;^1^* Miki Uchino, MD, PhD;^1^ Yoko Ozawa, MD, PhD;^1^ Mariko Sasaki, MD, PhD;^1,3,4^ Kazumasa Yamagishi, MD, PhD;^5,6^ Hiroyasu Iso, MD, PhD, MPH;^7^ Kazuo Tsubota, MD, PhD;^1^ and Shoichiro Tsugane, MD, PhD^2^

1. Department of Ophthalmology, Keio University School of Medicine, Tokyo 160-8582, Japan
2. Epidemiology and Prevention Group, Center for Public Health Sciences, National Cancer Center, Tokyo 104-0045, Japan
3. Tachikawa Hospital, Tokyo 190-8531, Japan
4. National Institute of Sensory Organs, National Tokyo Medical Center, Tokyo 152-8902, Japan
5. Department of Public Health Medicine, Faculty of Medicine, and Health Services Research and Development Center, University of Tsukuba, Ibaraki 305-8575, Japan
6. Ibaraki Western Medical Center, Ibaraki 308-0813, Japan
7. Department of Social and Environmental Medicine, Osaka University Graduate School of Medicine, Osaka 565-0871, Japan

***** Correspondence: [yukikenya114@gmail.com](about:blank), Tel: +81-3-5363-3821 (K.Y.); [nsawada@ncc.go.jp](about:blank); Tel: +81-3-3547-5201, (EXT. 3336) (N.S.)

**Supplementary Table S1. Distribution of intraocular pressure (IOP) in the right eye by age group.**

|  | Intraocular pressure, mmHg | | | | | | | |
| --- | --- | --- | --- | --- | --- | --- | --- | --- |
|  | All | |  | Men | |  | Women | |
| Age, years | N | Mean (SD) |  | N | Mean (SD) |  | N | Mean (SD) |
| All ages | 6786 | 14.0 (2.8) |  | 2773 | 13.7 (2.8) |  | 4013 | 14.1 (2.8) |
| 40–49 | 771 | 14.5 (2.8) |  | 279 | 14.2 (2.8) |  | 492 | 14.6 (2.8) |
| 50–59 | 1053 | 14.3 (2.9) |  | 336 | 14.1 (2.9) |  | 718 | 14.3 (2.9) |
| 60–69 | 3003 | 14.1 (2.8) |  | 1176 | 13.9 (2.8) |  | 1826 | 14.2 (2.8) |
| 70–79 | 1742 | 13.5 (2.8) |  | 853 | 13.4 (2.9) |  | 889 | 13.7 (2.6) |
| 80 and older | 217 | 13.1 (2.5) |  | 129 | 12.9 (2.5) |  | 88 | 13.4 (2.5) |

IOP, intraocular pressure; SD, standard deviation.
